# Single ZnO Nanowire-Based Gas Sensors to Detect Low Concentrations of Hydrogen

**DOI:** 10.3390/s151229816

**Published:** 2015-12-04

**Authors:** Marlene N. Cardoza-Contreras, José M. Romo-Herrera, Luis A. Ríos, R. García-Gutiérrez, T. A. Zepeda, Oscar E. Contreras

**Affiliations:** 1 Centro de Nanociencias y Nanotecnología, Universidad Nacional Autónoma de México, Baja California 22800, Mexico; jmromo@cnyn.unam.mx (J.M.R.-H.); trino@cnyn.unam.mx (T.A.Z.); edel@cnyn.unam.mx (O.E.C.); 2 Centro de Investigación Científica y de Educación Superior de Ensenada, Ensenada, Baja California 22860, Mexico; lrios@cicese.mx; 3 Departamento de Investigación en Física, Universidad de Sonora, Hermosillo 83000, Mexico; rgarcia@cifus.uson.mx

**Keywords:** nanowire, sensors, hydrogen, SEM-FIB, nano-fabrication, devices

## Abstract

Low concentrations of hazardous gases are difficult to detect with common gas sensors. Using semiconductor nanostructures as a sensor element is an alternative. Single ZnO nanowire gas sensor devices were fabricated by manipulation and connection of a single nanowire into a four-electrode aluminum probe *in situ* in a dual-beam scanning electron microscope-focused ion beam with a manipulator and a gas injection system in/column. The electrical response of the manufactured devices shows response times up to 29 s for a 121 ppm of H_2_ pulse, with a variation in the nanowire resistance appreciable at room temperature and at 373.15 K of approximately 8% and 14% respectively, showing that ZnO nanowires are good candidates to detect low concentrations of H_2_.

## 1. Introduction

Conductometric gas sensors are electronic devices with a simple structure where their operation principle is based on the variation of electrical conductance of their sensor element; this can occur due to the electron exchange between the surface and conduction band as the result of oxidation/reduction when its surface is exposed by chemical gases. Semiconductors are one of the most commonly used materials for sensor elements, since naturally, in ambient air, a thin layer of native oxide is formed on a semiconductor’s surface, and in the presence of a reducing gas, the interaction of oxygen-adsorbed species on its surface and the objective gas molecules causes the chemical reduction of its surface. This phenomenon modifies the amount of active charge carriers in the semiconductor and, hence, its conductance.

Most conventional gas sensors are fabricated with microscopic sensor elements, which are unable to detect very low concentrations (parts per million, pmm) of gases. Nowadays, mandatory safety regulations for handling harmful and hazardous chemicals for human beings and our surrounding environments strictly demand faster and highly sensitive sensors. 

Semiconductor nanostructures as wires offer a great alternative to this problem, mainly due to their limited size and cylindrical geometry. ZnO is an n-type semiconductor, and when O_2_ molecules are adsorbed on a ZnO nanowire surface, they extract electrons from the conduction band and trap them at the semiconductor surface. This will lead to an electron depletion layer, and then there is no conduction on the semiconductor surface, and the remaining free charge carriers are concentrated in the bulk of the nanowire (conduction channel). Under the presence of a reducing gas, the depletion layer disappears due to the release of the electrons back into the nanowire, thus expanding the conduction channel, resulting in an increment of the nanowire conductance. The conduction in nanowires greatly depends on the density of trapped charges on their surface or the release of these charges back into the nanostructure. Specifically, ZnO has attracted considerable interest in electronic and photo-electronic devices such as UV/visible photodetectors and active components in solar cells and gas sensors [1,2,3,4,5,6]. ZnO is a direct wide-band-gap semiconductor (3.37 eV at RT), naturally grown as an n-type semiconductor, that according to the literature has an intrinsic behavior that results from intrinsic crystal defects such as oxygen vacancies and/or zinc interstitials. Materials with this feature are good candidates in the fabrication of fast-response and highly sensitive gas sensor devices for reducing atmosphere when n-type nanostructures are used as sensor elements.

The sensitivity of gas sensors can be highly dependent on temperature. Tien *et al.* [7] analyzed the hydrogen-sensing of multiple ZnO nanorods with Pt coating capable of detecting 500 ppm of hydrogen at room temperature. A higher temperature was used by Tien *et al.* in single-crystal ZnO nanowires coated with SnO_2_ showing a strong sensitivity to 500 ppm of H_2_ in N_2_ at 673.15 K [8].

Recently, Ranwa *et al.* [9] fabricated a Au/ZnO NRs/Si/ZnO NRs/Au Schottky junction-based nanosensor at different temperatures with 1% and 5% hydrogen environment and results showed that the sensitivity increases from 11% to 67% with temperature increasing from 323.15 K to 423.15 K, and they also showed a response of 14 s for 1% at an operating temperature of 373.15 K.

In this work we studied the conductometric response (electrical resistance) of single ZnO nanowires tested as gas sensor elements in order to explore their capability to detect very low concentrations of H_2_ at room temperature and at 373.15 K.

## 2. Experimental Section

Gas sensor devices were fabricated using ZnO nanowires electrically connected to a four-electrode aluminum (Al) probe. A typical photolithography process was applied to Si/SiO*_x_*/Al substrates to produce the patterned Al probes. Si/SiO*_x_*/Al layer structures were grown by a deposition of an Al thin layer on SiO_x_ thermally grown on silicon (Si) wafers. The ZnO nanowires were synthesized on Si substrates by chemical vapor deposition as described previously by Garcia-Gutierrez *et al.* [10]. The electrical connection process of each single nanowire over the Al probes was carried out *in-situ* in a dual-beam SEM-FIB (Jeol JIB-4500) equipped with a gas injection system (GIS) to perform Pt deposits and an Omniprobe Autoprobe 200.2 manipulator. Three single ZnO nanowire gas devices were fabricated using nanowires with diameters of 800, 700 and 500 nm, which are labeled M_1_, M_2_ and M_3_, respectively. I–V curves for all devices were acquired by electrical measurements carried out with an ECOPIA HMS-5000 Measurement System under room conditions, atmospheric-pressure and temperature conditions. Sensor devices were tested in a homemade gas chamber capable of performing time-dependent resistance measurements under well-controlled H_2_ atmospheres and temperatures up to 423.15 K. Very low H_2_ concentration atmospheres were achieved by carefully mixing dry air and 1% H_2_-argon balanced gases.

Later, gas sensor devices were introduced into the homemade gas chamber to evaluate their performance for detecting low H_2_ concentrations. Electrical resistance was now temporally monitored as a function of H_2_ concentration inside the chamber. The performance experiments are carried out using a continuous flow of dry air as a gas reference with a short pulse of a well-controlled H_2_ concentration.

Response is considered as the resistance variation percentage and was calculated through the next equation:

(1) $$∆R=[\frac{\left(R_{a}-R_{g}\right)}{R_{a}}]100$$

where *R_a_* and *R_g_* are the ZnO nanowire resistances before and during the H_2_ pulse, respectively.

## 3. Results and Discussion

**Figure 1:**
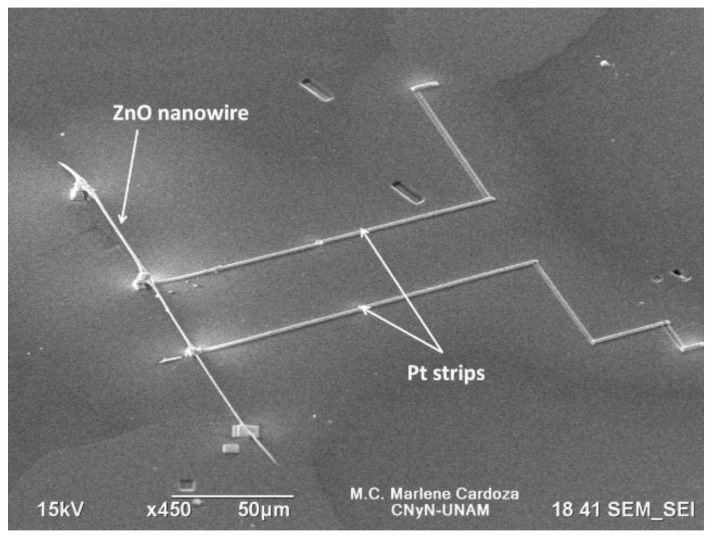
SEM image of fabricated single ZnO nanowire gas sensor device (M_1_).

Figure 1 shows a SEM image of the fabricated M_1_ gas sensor device displaying the ZnO nanowire connected to the Al probe through Pt strips deposited by the GIS.

**Figure 2:**
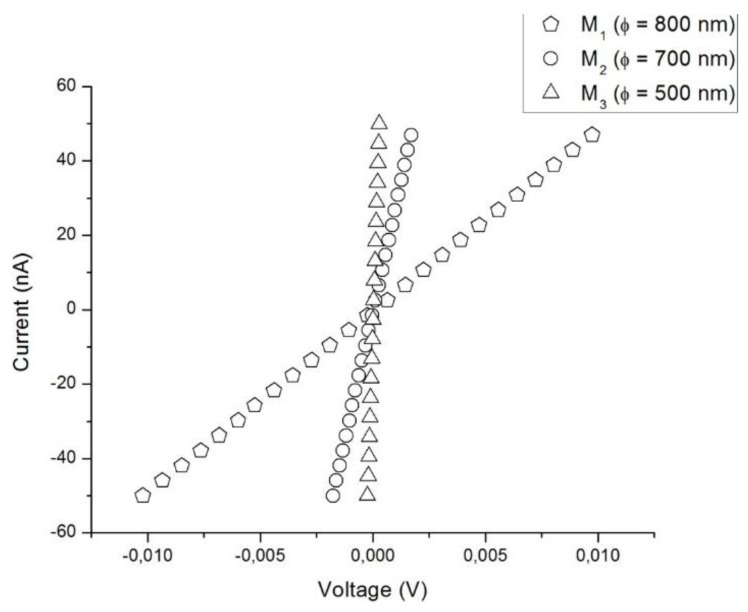
I–V characteristics of single ZnO nanowire devices showing an ohmic behavior.

Figure 2 shows the characteristic I–V curves measured for each sensor with an ohmic behavior clearly evident in the current range of —50 to +50 nA. As we can appreciate, there is a strong dependence on the nanowire diameter in the I–V characteristics. Since nanowires have a cylindrical shape, their electrical resistance can be estimated by Equation (2).

(2) $$R=\rho \;\frac{4l}{\pi D_{2}}=\frac{1}{\text{neμ}}\;\frac{4l}{\pi D_{2}}$$

where *R* is the nanowire electrical resistance, *ρ* is the nanowire electrical resistivity, *l* is the inner electrode distance, *µ* is the electronic mobility, and *D* is the nanowire diameter. According to the above equation, electrical resistance of the nanowires should have a major value as the diameter decreases. Nevertheless, this behavior would correspond to a passive electrical resistance where no changes in its electron density (n) are expected. Electrical resistances of single ZnO nanowires obtained from I–V curves shown in Figure 2 were 2.05 × 10^5^ Ω, 3.58 × 10^4^ Ω, and 4.38 × 10^3^ Ω for M_1_, M_2,_ and M_3_, respectively. Considering that I–V measures were performed at room conditions, the ZnO nanowires have trapped charges on their surface causing electrical resistance increments. Then ZnO electrical resistance turns out to be active and dependent on the electron density. This effect can be more evident as the diameter of the nanowire reduces and the resistance is more affected by surface phenomena.

**Figure 3:**
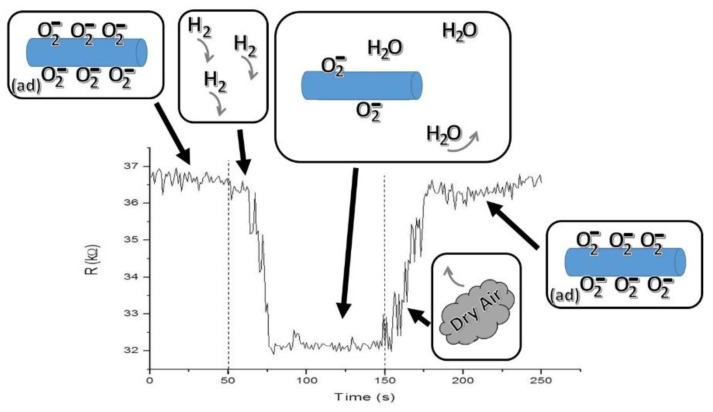
Sensing mechanism scheme occurring in one of the semiconductor devices, through the resistance change of M_2_ at a pulse concentration of 121 ppm of H_2_ at 373.15K.

Figure 3 consists of a scheme showing the proposal detection mechanism for a 100 s H_2_ pulse at a 121 ppm concentration in the M_2_ device at 373.15 K. In the first stage, the ZnO nanowire has oxygen ions trapped on its surface; then, since ZnO is an n-type semiconductor, the above causes the appearance of a charge depletion region next to the nanowire surface. ZnO nanowire has nanoscale dimensions and a limited conduction channel, and it can be assumed that a large amount of its carriers are trapped in surface states. In the second stage, a pulse with a concentration of 121 ppm of H_2_ was introduced into the homemade gas chamber. Hydrogen is very reactive with the oxygen ions adsorbed on the semiconductor nanowire surface, becoming vapor water as a product (normally carried out by the dry air H_2_ flow), and is consequently released back to the surface-trapped electrons. This interaction results in an increment of the majority charge carriers in the nanowire and, hence, a decrease from 36.3 to 31.8 kΩ of the nanowire resistance in a time of 27 s. This demonstrates that even a low concentration H_2_ pulse of 121 ppm can induce a measurable change in the nanowire resistance. In the last stage, the depletion region is rebuilt when the H_2_ gas pulse is removed, maintaining only the gas reference and leading again to gradual nanowire surface oxidation (until the nanowire resistance reaches at least 0.9 R_a_) and getting a recuperation time of 28 s.

**Figure 4:**
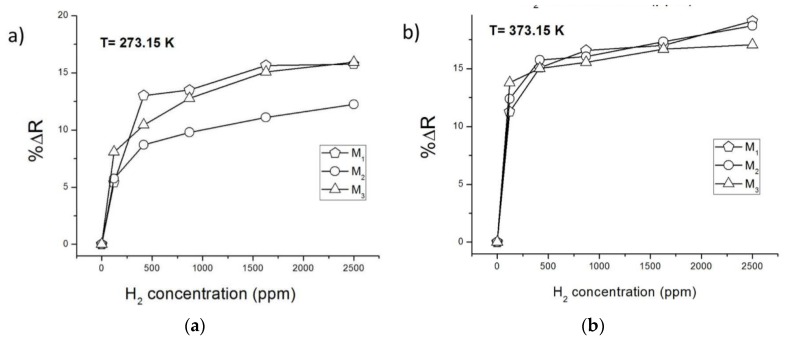
ZnO nanowire gas sensor devices percentage resistance changes for different H_2_ concentrations pulses. (**a**) At room temperature; (**b**) at 373.15 K.

Figure 4a,b show the ZnO nanowire resistance variations for the different H_2_ pulses at room temperature and at 373.15 K, respectively. It is observed at both temperatures that, at the lowest H_2_ concentration tested (121 ppm), the calculated %ΔR is greater for the ZnO nanowire of smaller diameter (M_3_), thus impacting the higher level of detection of the sensor element. This result is consistent considering that the smaller the diameter, the smaller the conduction channel of the nanowire, and hence, the greater the influence of the electrons released back from its surface on the resistance of the nanowire. Moreover, it is also noted that the major change of resistance was at the lowest concentration for the three devices; meanwhile, at high concentrations (> 417 ppm), increments of %ΔR of the nanowires are minimal (0.5%–2.5%). This shows that ZnO nanowires can detect very low concentrations of H_2_ and it is likely that the nanowire surface is getting depleted of oxygen at elevated concentrations of H_2_.

Finally, all gas sensor devices show higher %ΔR at 373.15 K, obtaining even a significant resistance variation of the major nanowire diameter device (M_1_) in response to a low H_2_ concentration pulse of 121 ppm. It is also demonstrated that the resistance variation at 373.15 K (Figure 4b) has a more stepped shape than at 300.15 K (Figure 4a), showing a greater uniformity of resistance variation percentage values between the fabricated devices.

## 4. Conclusions

In summary, we have shown that single ZnO nanowires–based H_2_ sensors are an alternative for detecting low concentrations of H_2_ due to their limited conduction channel that allows even a low concentration of injected electrons to produce a measurable change in their resistance.
